# Photosensitizer‐Anchored 2D MOF Nanosheets as Highly Stable and Accessible Catalysts toward Artemisinin Production

**DOI:** 10.1002/advs.201802059

**Published:** 2019-04-09

**Authors:** Ying Wang, Liang Feng, Jiandong Pang, Jialuo Li, Ning Huang, Gregory S. Day, Lin Cheng, Hannah F. Drake, Ye Wang, Christina Lollar, Junsheng Qin, Zhiyuan Gu, Tongbu Lu, Shuai Yuan, Hong‐Cai Zhou

**Affiliations:** ^1^ College of Chemistry Tianjin Normal University Tianjin 300387 China; ^2^ Key Laboratory of Advanced Energy Materials Chemistry (Ministry of Education) Nankai University Tianjin 300071 China; ^3^ Department of Chemistry Texas A&M University College Station TX 77843 USA; ^4^ Institute of New Energy Materials & Low Carbon Technology School of Material Science & Engineering Tianjin University of Technology Tianjin 300384 China; ^5^ College of Chemistry and Materials Science Nanjing Normal University Nanjing 210023 China; ^6^ Department of Materials Science and Engineering Texas A&M University College Station TX 77842 USA

**Keywords:** artemisinin, metal–organic frameworks, photochemical synthesis

## Abstract

2D metal–organic frameworks (2D‐MOFs) have recently emerged as promising materials for gas separations, sensing, conduction, and catalysis. However, the stability of these 2D‐MOF catalysts and the tunability over catalytic environments are limited. Herein, it is demonstrated that 2D‐MOFs can act as stable and highly accessible catalyst supports by introducing more firmly anchored photosensitizers as bridging ligands. An ultrathin MOF nanosheet‐based material, Zr‐BTB (BTB = 1,3,5‐tris(4‐carboxyphenyl)benzene), is initially constructed by connecting Zr_6_‐clusters with the tritopic carboxylate linker. Surface modification of the Zr‐BTB structure was realized through the attachment of porphyrin‐based carboxylate ligands on the coordinatively unsaturated Zr metal sites in the MOF through strong Zr‐carboxylate bond formation. The functionalized MOF nanosheet, namely PCN‐134‐2D, acts as an efficient photocatalyst for ^1^O_2_ generation and artemisinin production. Compared to the 3D analogue (PCN‐134‐3D), PCN‐134‐2D allows for fast reaction kinetics due to the enhanced accessibility of the catalytic sites within the structure and facile substrate diffusion. Additionally, PCN‐134(Ni)‐2D exhibits an exceptional yield of artemisinin, surpassing all reported homo‐ or heterogeneous photocatalysts for the artemisinin production.

## 1 Introduction

Since the isolation of graphene in 2004, 2D materials have progressed rapidly owing to their unique anisotropic chemicals, electronics, mechanical properties, and mass transport properties.^1^ Within the last decade there has been a rapid increase in the types of 2D materials available. In addition to graphene, researchers have developed other materials including hexagonal boron nitride compounds, transition metal dichalcogenides, transition metal chalcogenides, metal oxides, metal carbides, layered silicates, and layered zirconium phosphates/phosphonates.^2^, ^3^, ^4^, ^5^, ^6^, ^7^ Amongst these materials, α‐zirconium phosphate (α‐ZrP) represents an interesting class of 2D layered materials.^7^ Although ZrPs do not have atomic level thickness like graphene, their interlayer spaces are surrounded by −OH groups and thus can be easily modified through cation exchange or through surface functionalization. This is similar to functionalization seen in organic silanes, isocyanate, and epoxides.^8^ Therefore, ZrP have been widely investigated for applications in ion exchange, catalysis, and drug delivery.^9^ Nevertheless, as an inorganic material, the structural tunability of ZrPs is still relatively limited.

We propose that metal–organic frameworks (MOFs) can act as inorganic‐organic hybrid analogues of ZrP with enhanced structural diversity and functional tunability. MOFs are extended network structures constructed from inorganic metal nodes and organic ligands, typically resulting in 3D materials with inherent porosity.^10^, ^11^ Although 3D MOFs have been intensively studied for a wide range of applications including gas storage, separation, sensing, catalysis, and biomedicine,^12^, ^13^, ^14^, ^15^, ^16^ there remains very few application‐based research studies on 2D MOFs. 2D MOFs represent a significant improvement over inorganic 2D materials due to their capability of functionality through the judicious selection of inorganic nodes and/or organic linkers. Amongst the few examples found in the literature, Yang and co‐workers demonstrated the application of exfoliated 2D MOF nanosheets as molecular sieving membranes.^17^ In another report, Wang and co‐workers incorporated metallo‐terpyridyl catalytic centers into a surface modified 2D MOF layer for the selective oxidation of C—H bonds.^18^ We aim to extend the current direction of 2D MOF catalysis by combining the advantages of both heterogenous and homogenous systems. Various homogenous catalysts with defined structures can be incorporated into 2D MOF through the modification of metal nodes and/or organic ligands. Meanwhile, 2D nanosheets with inherent porosity facilitate the diffusion of substrates and enhance the accessibility of active sites in the material.

With these strategies in mind, a previously reported 2D MOF based on Zr_6_ clusters and the tritopic carboxylate ligand 1,3,5‐tris(4‐carboxyphenyl)benzene (BTB), was selected as a catalyst support for our study.^19^, ^20^, ^21^ The resulting structure, Zr‐BTB, contains Zr_6_ clusters that are hexacoordinate in the equatorial plane, thus leaving six pairs of terminal −OH−/H_2_O above and below the layer for functionalization of the material. The terminal −OH−/H_2_O in the structure can be replaced by carboxylate ligands, providing a facile approach for surface modification. Therefore, Zr‐BTB can be perceived as a MOF analogue of inorganic ZrP with enhanced tunability. In this work, 2D Zr‐BTB nanosheets with controlled thicknesses were synthesized by adjusting the interlayer van der Waals interactions. Porphyrin‐based ligands were subsequently anchored on the surface of the Zr‐BTB nanosheets by replacing the terminal −OH−/H_2_O on the Zr_6_ clusters. The resulting 2D material, PCN‐134‐2D, was utilized as a photocatalyst for the oxidation of dihydroartemisinic acid to produce artemisinin. When compared to the 3D counterpart (PCN‐134‐3D), the 2D nanosheets (PCN‐134‐2D) demonstrated improved reaction kinetics. This improvement was due to the more readily accessible catalytic centers that the high dispersibility of the individual nanosheets provided over that of the 3D PCN‐134 structure.

## 2 Results and Discussions

### 2.1 Design of 2D MOFs

**Figure 1:**
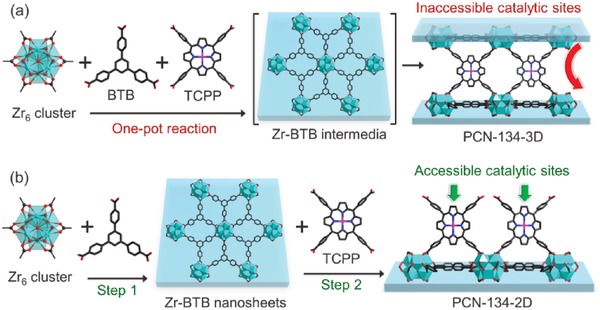
a) Schematic representation showing the one‐pot synthesis of PCN‐134‐3D. b) Stepwise synthesis of PCN‐134‐2D nanosheets with accessible catalytic sites.

Among the limited number of 2D Zr‐MOFs, Zr‐BTB stands out as a suitable catalyst support. This MOF was initially reported by Sun and co‐workers as interpenetrated nets, and was further studied by the Matzger and Zhao groups as a 2D material.^19^, ^20^, ^21^ Layered Zr‐BTB is formed by linking six‐connected Zr_6_ clusters with the tritopic Zr‐BTB linker into a (3,6)‐connected network. This arrangement allows for the formation of a coordinately unsaturated Zr_6_ cluster in the MOF. The coordinatively unsaturated Zr_6_ clusters endow unique tunability to the Zr‐BTB structure, with the terminal −OH−/H_2_O ligands being replaced by carboxylate ligands. This unique tunability allows for the functionalization of the Zr‐BTB nanosheets.^20^ Our previous work has shown that the Zr‐BTB layers can be extended into a 3D network by utilizing the tetratopic porphyrin‐based linker, tetrakis(4‐carboxyphenyl)porphyrin (TCPP), as a pillaring ligand, forming a layer‐pillar‐type framework (PCN‐134‐3D, **Figure** **1**a).^22^ Because of the limited pore size of PCN‐134‐3D (1.1 nm), large substrates are usually excluded from the pore cavity, limiting its application in catalysis.

This problem can be overcome by reducing the 3D MOF to a 2D regime. To obtain PCN‐134‐2D, a stepwise synthetic method was designed. The traditional synthesis of PCN‐134‐3D was carried out in a one‐pot reaction without the isolation of the Zr‐BTB intermediate (Figure 1a). In contrast, the stepwise synthesis of PCN‐134‐2D involves the initial isolation of ultrathin Zr‐BTB nanosheets and the subsequent modification of Zr‐BTB nanosheets with TCPP functionalities (Figure 1b). The resulting PCN‐134‐2D has highly accessible porphyrin centers tethered to the surface of the nanosheets. For comparison, the one‐pot synthesis of PCN‐134‐2D through the exfoliation of PCN‐134‐3D was attempted, but was unsuccessful. This was most likely because the strong Zr‐carboxylate bonds connecting the Zr‐BTB layers prevented nanosheet isolation in this procedure.

### 2.2 Control the Thickness of Zr‐BTB

When modulating reagents, such as monocarboxylic acids, were used during the MOF synthesis, the terminal carboxylates coordinated to the out of plane binding sites on the Zr_6_ clusters. It has been previously demonstrated that these terminal ligands can be utilized to alter the interlayer distances.^20^ Traditional solvothermal syntheses using ZrCl_4_, H_3_BTB, and modulating acids resulted in closely packed Zr‐BTB bulk materials. This is attributed to the strong van der Waals interactions between terminal carboxylate ligands from adjacent layers. One way to weaken the interlayer interactions is to reduce the amount of terminal carboxylate ligands. This can be realized by adding water during the MOF synthesis to partially replace the terminal carboxylate ligands with terminal −OH^−^/H_2_O ligands. Alternatively, the interlayer interactions can be changed through the alteration of the functionalities in the modulating reagents. The substituents on the terminal carboxylate ligands affect the interlayer interactions, modulating the thickness of the MOF particles.

**Figure 2:**
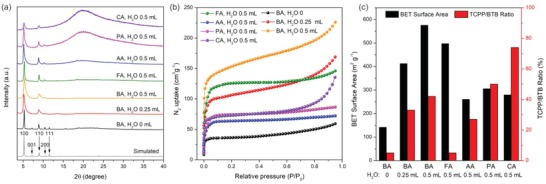
a) PXRD patterns of Zr‐BTB sheets produced under different synthetic conditions. The simulated pattern was based on structural models in a hexagonal lattice with *P*‐31*m* space group (*a* = *b* = 19.595 Å, *c* = 12.264 Å). b) N_2_ adsorption isotherms of Zr‐BTB sheets at 1 bar, 77 K. c) Comparison of BET surface areas and TCPP/BTB ratios of Zr‐BTB sheets synthesized under different conditions.

To investigate the effect of water addition and terminal carboxylate ligands, a series of control experiments were conducted wherein different types of modulating acid and various amount of water were added to the reaction mixture for the synthesis of the Zr‐BTB material. Generally, a mixture of ZrCl_4_ (10 mg), Zr‐BTB (10 mg), DMF (3 mL), terminal carboxylic acid ligand, and H_2_O were heated at 120 °C for 48 h to obtain the Zr‐BTB product. The resulting product was analyzed by powder X‐ray diffraction (PXRD) and scanning electron microscopy (SEM) to determine the structure and layer thickness. The addition of water significantly hindered the packing of the Zr‐BTB layers as indicated by the gradually reduction in diffraction intensity along the *c*‐direction in the PXRD pattern. When benzoic acid (BA) was used as the modulating agent, the peaks corresponding to the 001 and 111 crystal faces disappeared upon the addition of water to the reaction (**Figure** **2**a and Figure S5,Supporting Information). A similar phenomenon was also observed when other modulators such as formic acid (FA), acetic acid (AA), propionic acid (PA), and caproic acid (CA) were utilized.

The modulating carboxylic acid units not only changed the thickness of the Zr‐BTB sheets but also affected the crystallinity of the product. Generally, the addition of modulating carboxylic acids with bulky substituents expands the interlayer distances and prevents interlayer packing. For example, FA gave rise to thicker Zr‐BTB nanosheets as indicated by the strong (001) and (111) diffraction peaks in the PXRD (Figure S1, Supporting Information), whereas these peaks were not observed when bulkier modulating carboxylic acids were used (Figures S2–S4, Supporting Information). During the MOF synthesis, the modulating carboxylate acid linkers competitively coordinate with the metal cations, slowing down the crystal growth and allowing for the formation of highly crystalline products. The interaction between the metal cations and modulating carboxylates become diminished when bulky substituents are used due to steric hindrances, resulting in a weaker modulating effect. Therefore, bulky carboxylic acids such as PA and CA produced low crystallinity Zr‐BTB products (Figures S3 and S4, Supporting Information). This is in line with the BET surface areas estimated by N_2_ adsorption isotherms. Samples obtained using AA, PA, and CA showed relatively low surface areas (Figure 2b,c and Figure S6, Supporting Information). This was attributed to the poor crystallinity of the product MOF. When BA was adopted as a modulator, the total N_2_ uptakes and surface area became greater upon the addition of water (Figure 2b and Figure S7, Supporting Information). This is ascribed to the partial removal of the terminal carboxylate ligands and a reduction in layer thickness which exposed otherwise inaccessible surfaces between the densely packed layers. The particle size and layer thickness for the structures were further monitored by SEM (Figure S8, Supporting Information). The use of FA as a modulating agent led to the formation of thick hexagonal plates, whereas AA, PA, and CA modulators resulted in particles with irregular morphologies. When BA was used for modulation, the Zr‐BTB nanosheets were uniform. A delicate balance between layer thickness and crystallinity can be reached by using BA as the modulating reagent followed by the addition of 0.5 mL water, producing thin Zr‐BTB nanosheets with regular morphology and high surface area. Detailed reaction conditions for the synthesis of the Zr‐BTB nanosheets are listed in Table S1 in the Supporting Information. Based on the initial results with BA modulation followed by water treatment, we opted to use the optimized 0.5 mL of water for testing with other modulators.

### 2.3 Surface Functionalization of Zr‐BTB

Porphyrin catalysts were attached to the Zr‐BTB nanosheets by incubating the as‐synthesized Zr‐BTB samples in solutions of DMF and TCPP at 100 °C. The TCPP uptakes into the structure, represented by TCPP/BTB ratios, were determined by the ^1^H‐NMR spectra of the digested samples (Figure 2c). TCPP are selectively bound to the surface of the Zr‐BTB layers through the TCPP carboxylate groups. This selectivity was apparent as the interlayer spaces in the parent structure were too small for the intercalation of TCPP molecules. In general, the TCPP uptakes values are directly related to the layer thickness in the structures. For example, the TCPP uptake increases upon the addition of H_2_O into the BA modulated samples. This result corresponded to a reduced layer thickness relative to the exposed surfaces in the structure. It should be noted that Zr‐BTB samples with low crystallinity usually exhibited high concentrations of missing BTB defects, which explains the high TCPP/BTB ratio upon TCPP adsorption in these samples. Indeed, the lower mass loss upon linker decomposition (≈500 °C) for the Zr‐BTB samples modulated by AA (12%), PA (11%), and CA (20%) were comparable to those of the samples modulated by FA (31%) and BA (32%). This was determined through thermogravimetric analysis (TGA) and indicated a greater degree of missing BTB defects in the structures (Figure S9, Supporting Information). Based on the layer thickness, crystallinity, surface areas, and TCPP uptakes, the Zr‐BTB nanosheets synthesized using BA as a modulator followed by 0.5 mL water were selected for further studies. PCN‐134‐2D was obtained by the TCPP modification of aforementioned Zr‐BTB nanosheets.

### 2.4 Structure of PCN‐134‐2D

**Figure 3:**
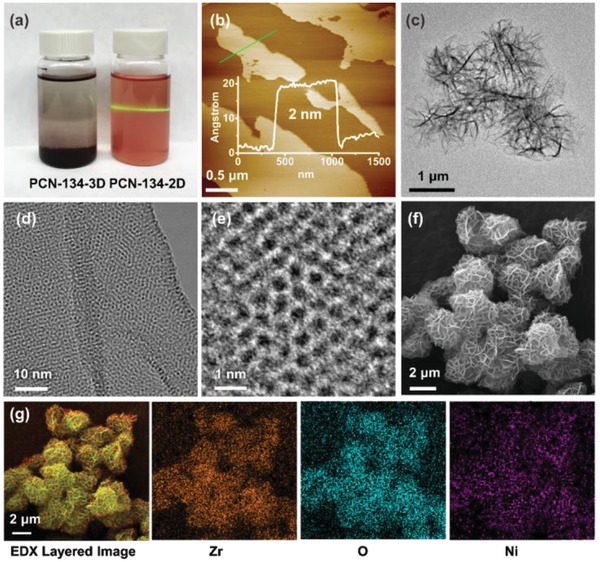
a) Photographs of PCN‐134‐3D (left) and PCN‐134‐2D (right) dispersed in water, displaying the Tyndall effect. b) AFM image of PCN‐134‐2D nanosheets with corresponding height profiles. c) TEM image of PCN‐134‐2D nanosheets showing a hexagonal lattice. d,e) HR‐TEM images of PCN‐134‐2D nanosheets. f) SEM image of PCN‐134‐2D nanosheets. g) Elemental mapping by SEM/EDX for PCN‐134‐2D. The porphyrin centers are preoccupied by Ni.

The structure and morphology of PCN‐134‐2D was systematically studied. The Tyndall scattering of PCN‐134‐2D showed a stable colloidal suspension (**Figure** **3**a and Figure S10, Supporting Information). Experiments utilizing SEM, transmission electron microscopy (TEM), and atomic force microscopy (AFM) indicated free standing nanosheets up to several micrometers in size. Wrinkled sheets were observed from the TEM (Figure 3c), suggesting an ultrathin nature of the nanosheets. The hexagonal lattice was clearly shown in the HR‐TEM image. These results were in line with the Zr‐BTB structure predicted (Figure 3d,e). These wrinkled sheets were shown to aggregate on a larger scale than previously predicted, indicated by the SEM images (Figure 3f). The height of PCN‐134‐2D was measured to be ≈2.0 nm with slight variations, roughly corresponding to the thickness of one or two molecular layers (Figure 3b and Figure S11, Supporting Information). The morphology of Zr‐BTB nanosheets was not noticeably altered by the surface modification of TCPP as demonstrated by the similar SEM, TEM, and AFM images of Zr‐BTB and PCN‐134‐2D (Figures S11–S13, Supporting Information).

To investigate the distribution of TCPP within PCN‐134‐2D, the Ni‐porphyrin‐based ligand (TCPP‐Ni) was used for the synthesis of PCN‐134‐2D‐Ni. The Ni in the porphyrin center was intended to act as a probe indicating the position of the TCPP ligand using elemental mapping by SEM/energy‐dispersive X‐ray (EDX) analysis and TEM/EDX. Elemental mapping indicated an overlapped distribution of Zr and Ni, thus demonstrating that the TCPP linkers were evenly distributed throughout the Zr‐BTB layer (Figures S12 and S13, Supporting Information). Further evidence for the distribution of the TCPP ligands were provided by the N_2_ adsorption isotherms (Figure S14, Supporting Information). The Zr‐BTB sample before and after TCPP incorporation exhibits similar N_2_ adsorption isotherms and almost identical pore size distributions, suggesting that TCPP was attached to the surface of the Zr‐BTB layer without occupying the MOF cavities. The slightly reduced surface area and N_2_ total uptake is explained by the increased formula weight after TCPP incorporation.

**Figure 4:**
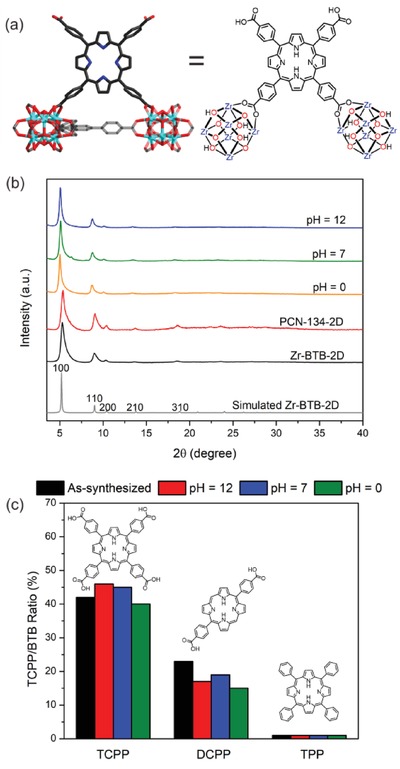
a) Proposed binding mode of TCPP in PCN‐134‐2D. b) PXRD of Zr‐BTB and PCN‐134‐2D treated by aqueous solutions with different pH values. c) Stability test of Zr‐BTB modified by TCPP, DCPP, and TPP treated by aqueous solutions with different pH values.

We propose that the TCPP was bridging a pair of adjacent Zr‐clusters within the PCN‐134‐2D (**Figure** **4**a), similar to the coordination mode observed in PCN‐134‐3D single crystals. Control experiment was carried out by truncating the carboxylate groups from a tetracarboxylate porphyrin‐based linker. Porphyrin molecules without carboxylates or with only two carboxylates showed only a weak interaction with the Zr‐BTB layer. In fact, these porphyrin molecules were easily removed through washing the structures with DMF. On the contrary, the tetracarboxylate TCPP linkers showed a much stronger interaction with the Zr‐BTB layer surviving treatment of a wide pH range in aqueous solution (Figure 4b). No obvious TCPP leaching was observed during the stability tests as indicated by UV–vis studies of the supernatants (Figure S15, Supporting Information). Based on the proposed TCPP binding modes, ^1^H‐NMR, and elemental analysis, the composition of PCN‐134‐2D was determined to be [Zr_6_O_4_(OH)_4_](OH)_4.2_(H_2_O)_4.2_(BTB)_2_(H_2_TCPP)_0.9_.

### 2.5 Enhanced Accessibility of Porphyrin Catalytic Center

**Figure 5:**
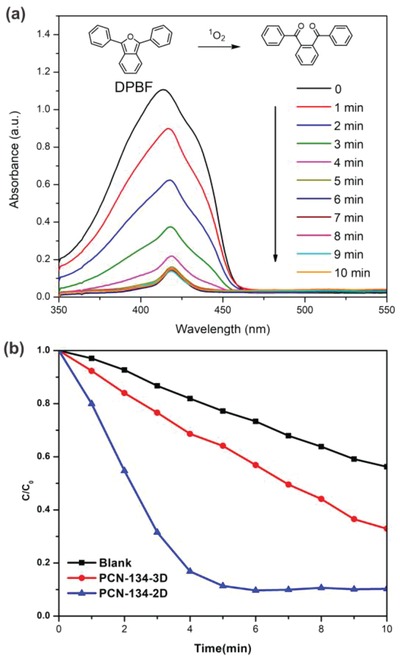
a) UV–vis absorption spectra of DPBF upon visible‐light irradiation with PCN‐134‐2D nanosheets. b) The degradation of DPBF using PCN‐134‐3D and PCN‐134‐2D as monitored by the absorbance decay at 410 nm.

Porphyrin derivatives have been widely applied as photocatalysts for ^1^O_2_ generation because of their high light harvesting efficiency.^23^, ^24^ Photogenerated ^1^O_2_ has a variety of potential applications in organic synthesis, wastewater treatment, and photodynamic therapy.^25^, ^26^, ^27^ Incorporating porphyrins into ultrathin 2D nanosheets creates highly accessible porphyrin sites for photocatalytic ^1^O_2_ generation. To determine the efficiency of this process, we utilized a well‐known ^1^O_2_ scavenger, 1,3‐diphenylisobenzofuran (DPBF) to monitor the ^1^O_2_ generation by the porphyrin‐based MOF catalysts. Upon visible‐light irradiation (λ > 420 nm) in the presence of PCN‐134‐2D, the absorption at 410 nm decreased, indicating the formation of ^1^O_2_ (**Figure** **5**a). The degradation rate of DPBF is much slower for the unfunctionalized Zr‐BTB due to the absence of the porphyrin photosensitizer. The 2D MOF nanosheet variant of PCN‐134 exhibited superior catalytic activity when compared with to the 3D counterpart (Figure 5b). This is attributed to the higher accessibility of the porphyrin sites in PCN‐134‐2D over that of the accessibility of the sites in PCN‐134‐3D. The small pore aperture and large particle size of PCN‐134‐3D severely limited the diffusion of DPBF into the MOF particles, resulting in slow reaction kinetics.

### 2.6 Photocatalytic Oxidation of Dihydroartemisinic Acid to Artemisinin

With preliminary result showing visible‐light sensitized production of ^1^O_2_ by PCN‐134‐2D nanosheets, we decided to further investigate PCN‐134‐2D nanosheets in organic synthesis application. Visible‐light sensitized generation of ^1^O_2_ is a key step for many organic transformations such as the semisynthetic production of artemisinin, an important antimalarial drug, from dihydroartemisinic acid.^28^, ^29^ Considering the fact that currently the most effective treatment against malaria is believed to be artemisinin, the World Health Organization recommends artemisinin‐based combination therapies (ACTs) as first‐line drugs. One effective approach to minimize the cost of artemisinin extraction or total synthesis is through the photochemical semisynthesis from biosynthetic precursors (artemisinic acid).^30^, ^31^ The reaction starts with the oxidation of dihydroartemisinic acid with ^1^O_2_ to yield the allylic hydroperoxide intermediate. The intermediate then undergoes a Hock cleavage to afford a ketone and aldehyde/enol nucleophile. Following this step, ^3^O_2_ is added to the reaction, and a final ring closure step is performed to give the final product, artemisinin.

The synthesis of artemisinin was conducted using PCN‐134‐2D nanosheets as a photocatalyst under visible light in the presence of O_2_. When PCN‐134‐2D nanosheets were adopted as a catalyst, almost full conversion was achieved within 1 h. The reaction was allowed to continue for 3 h, giving a conversion of 99% and a yield of 53% (**Table** **1**, entry 1). It should be noted that most photocatalysts give yields between 50% and 60% after optimization. These yields are attributed to the inherent limitations of the reactions themselves.^32^ The conversion rate of the PCN‐134‐3D catalytic system is noticeably slower than PCN‐134‐2D due to the limited pore sizes. This restriction hinders substrate diffusion toward the catalytic centers in the structures (Table 1, entry 2). It should also be noted that the nanosheets can be well dispersed in solution, forming a colloidal suspension. The incorporation of a suspension can further enhance the accessibility of the catalytic sites and facilitate further substrate diffusion. When compared to the molecular porphyrin catalysts, PCN‐134‐2D can tolerate a wider range of solvents, including CH_2_Cl_2_ and EtOH:H_2_O, without a significant change in the substrate conversion (Table 1, entry 5).

**Table 1:**
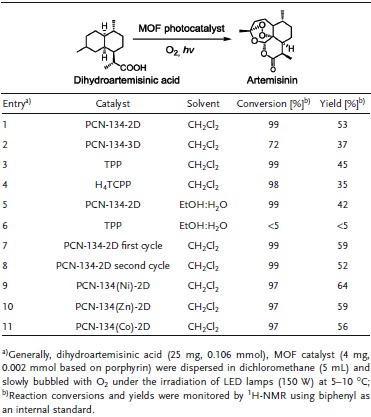
Synthesis of artemisinin from dihydroartemisinic acid using different photocatalysts

It should be noted that the molecular catalysts, TPP and H_4_TCPP, have a lower conversion rate as compared with the PCN‐134‐2D nanosheets, as these materials are not soluble in EtOH:H_2_O, nor do they form a true colloidal suspensions (Table 1, entry 3 and 6). Despite being a well dispersed suspension, PCN‐134‐2D can still be easily separated from the reaction mixture using filtration or centrifugation. The catalytic activity and crystallinity of PCN‐134‐2D is maintained after three cycles (Table 1, entry 7 and 8). To further improve the selectivity of the artemisinin, various metals (Ni, Zn, and Co) were incorporated into porphyrin linkers to accelerate ^1^O_2_ involved step. Remarkably, PCN‐134(Ni)‐2D exhibits much higher yield of artemisinin, 64%, which has surpassed all reported photocatalysts for the semisynthesis of artemisinin (Table 1; entry 9, 10, and 11). These results highlight the advantage of 2D MOFs catalysts as highly accessible and recyclable heterogeneous catalysts with tailored functionalities.

## 3 Conclusion

In conclusion, we have demonstrated that a 2D MOF, Zr‐BTB, can act as a suitable catalyst support. Surface modification of Zr‐BTB nanosheets with porphyrin ligands were shown to give rise to an efficient ^1^O_2_ photocatalyst for artemisinin production. The reaction kinetics of the 2D nanosheets (PCN‐134‐2D) and their 3D counterparts (PCN‐134‐3D) were compared, highlighting the enhanced substrate diffusion and catalyst accessibility of the 2D MOFs. Significantly, PCN‐134(Ni)‐2D exhibits highest yield of artemisinin among all reported homo‐ or heterogeneous photocatalysts for the artemisinin production. Considering the structural diversity and functional tunability of 2D MOFs, a variety of 2D MOF catalysts can be envisioned from the results of this work. Of most important significance, 2D MOF catalysts will bring new concepts to catalysis by blurring the distinction between homogeneous and heterogeneous catalysts while still preserving the desirable attributes of both the systems.

## Conflict of Interest

The authors declare no conflict of interest.

## Supporting information

Supplementary
