# Supplementary material for: Photosensitizer‐Anchored 2D MOF Nanosheets as Highly Stable and Accessible Catalysts toward Artemisinin Production
Source: Adv Sci (Weinh). 2019 Apr 9;6(11):1802059. doi: 10.1002/advs.201802059 (PMC6548987; doi:10.1002/advs.201802059)
Supplement: Supplementary file 1 — Supplementary [file ADVS-6-1802059-s001.pdf]

Copyright WILEY-VCH Verlag GmbH & Co. KGaA, 69469 Weinheim, Germany, 2019.

ADVANCED SCIENCE
Open Access

Supporting Information

for *Adv. Sci.,* DOI: 10.1002/advs.201802059

Photosensitizer-Anchored 2D MOF Nanosheets as Highly Stable and Accessible Catalysts toward Artemisinin Production

Ying Wang, Liang Feng, Jiandong Pang, Jialuo Li, Ning Huang, Gregory S. Day, Lin Cheng, Hannah F. Drake, Ye Wang, Christina Lollar, Junsheng Qin, Zhiyuan Gu, Tongbu Lu, Shuai Yuan,* and Hong-Cai Zhou**

Supporting Information

Photosensitizer-Anchored Two-Dimensional MOF Nanosheets as Highly Stable and Accessible Catalysts Toward Artemisinin Production

Shuai Yuan,[‡,§] Liang Feng,[‡,§] Ying Wang,*[,†,‡,‖,§] Jiandong Pang,[‡] Jialuo Li,[‡] Ning Huang,[‡] Gregory S. Day,[‡] Lin Cheng,[†] Hannah F. Drake,[‡] Ye Wang,[¶] Christina Lollar,[‡] Junsheng Qin,[‡] Zhiyuan Gu,[⊥] Tongbu Lu,[¶] and Hong-Cai Zhou*[,‡,#]

[†]College of Chemistry, Tianjin Normal University, Tianjin, 300387, China

[‡]Department of Chemistry, Texas A&M University, College Station, Texas 77843-3255, United States

[#]Department of Materials Science and Engineering, Texas A&M University, College Station, Texas 77842, United States

[‖]Key Laboratory of Advanced Energy Materials Chemistry (Ministry of Education), Nankai University, Tianjin 300071, China

[¶]Institute of New Energy Materials & Low Carbon Technology, School of Material Science & Engineering, Tianjin University of Technology, Tianjin 300384, China

[⊥]College of Chemistry and Materials Science, Nanjing Normal University, Nanjing, China

[§] These authors contributed equally to this work.

Corresponding Author

*wangying790601@163.com

*zhou@chem.tamu.edu

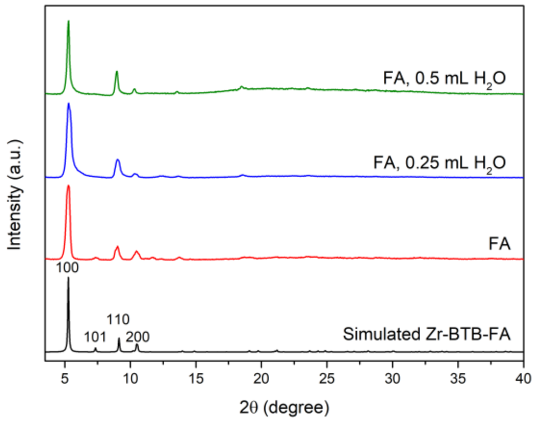

Figure S1. PXRD patterns of Zr-BTB synthesized by formic acid (FA).

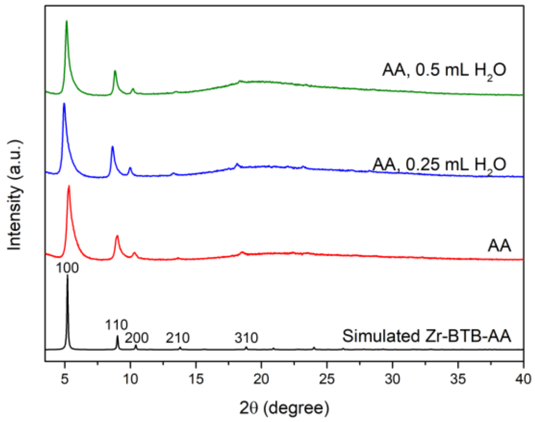

Figure S2. PXRD patterns of Zr-BTB synthesized by acetic acid (AA).

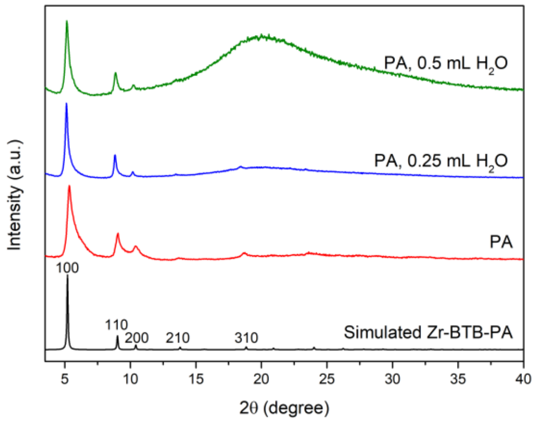

Figure S3. PXRD patterns of Zr-BTB synthesized using propanoic acid (PA) as a modulator.

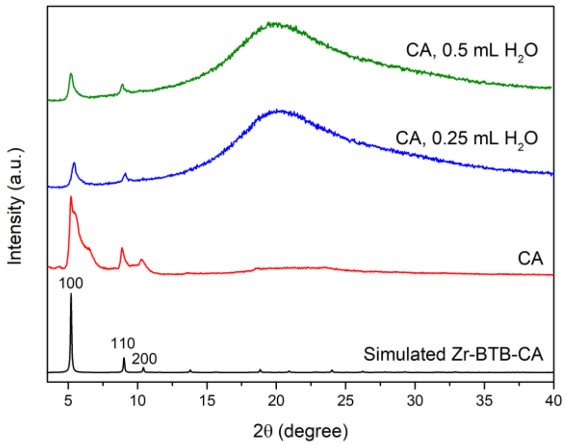

Figure S4. PXRD patterns of Zr-BTB synthesized by caproic acid (CA) used as a modulator.

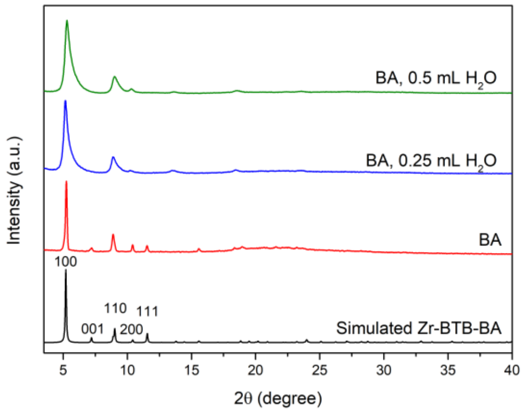

Figure S5. PXRD patterns of Zr-BTB synthesized by benzoic acid (BA) used as a modulator.

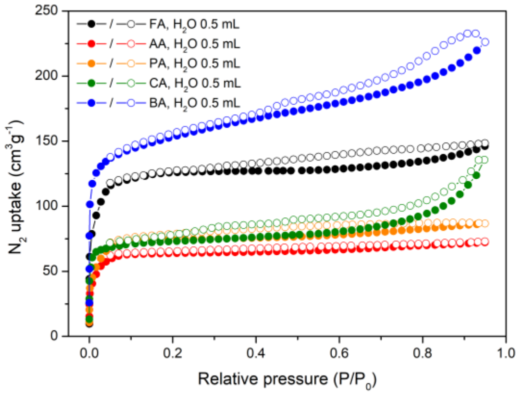

Figure S6. N_2 sorption isotherms of Zr-BTB at 77 K showing the effect of modulator identity on the total gas uptake.

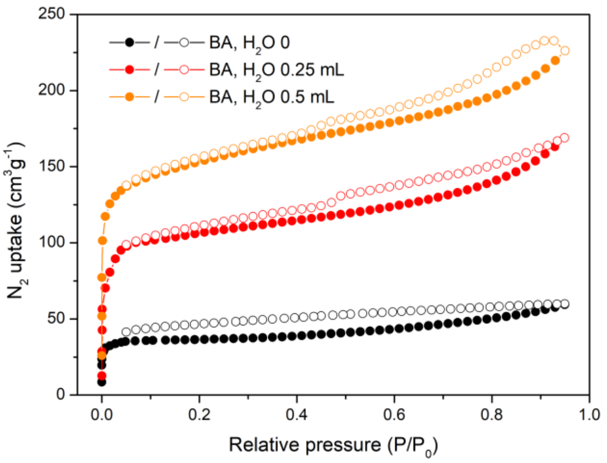

Figure S7. N_2 sorption isotherms of Zr-BTB at 77 K showing the effect of water hydrolysis on the benzoic acid modulated samples.

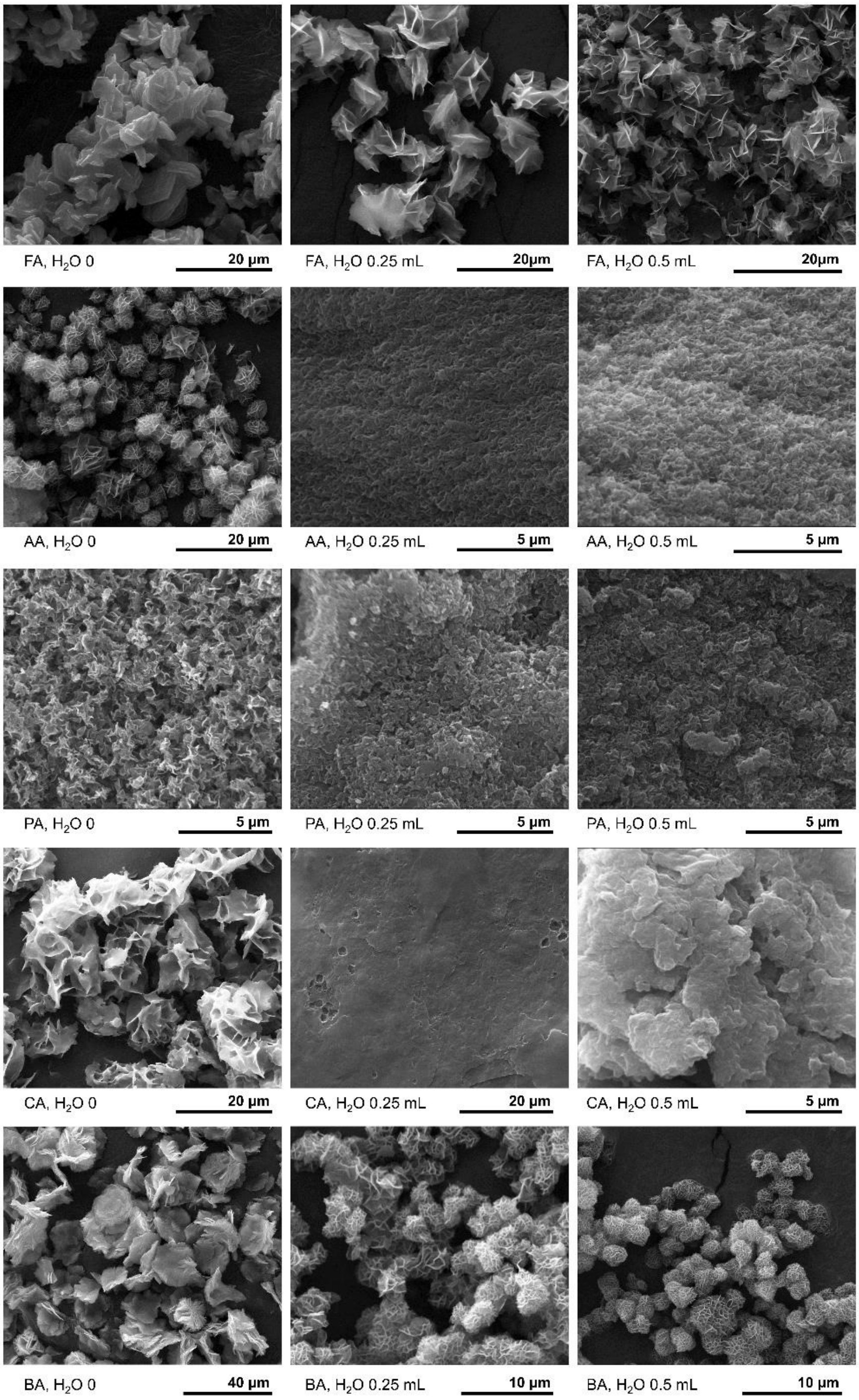

Figure S8. SEM images of Zr-BTB synthesized under different conditions showing the effect of modulator and water.

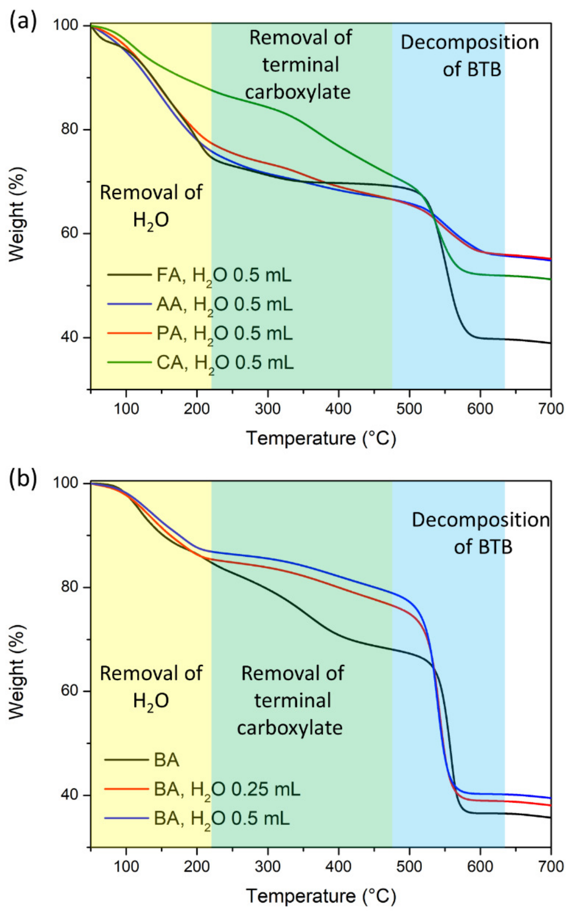

Figure S9. TGA of Zr-BTB synthesized using different modulating acids (a) and different amount of water (b).

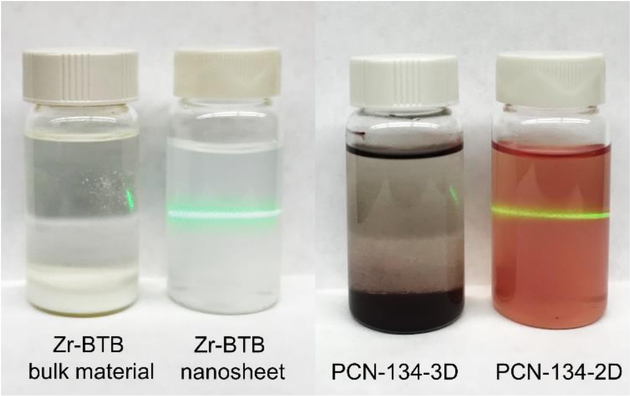

Figure S10. Photographs of Zr-BTB bulk material, Zr-BTB nanosheets, PCN-134-3D, and PCN-134-2D nanosheets dispersed in water. Only the nanosheets exhibit the Tyndall effect.

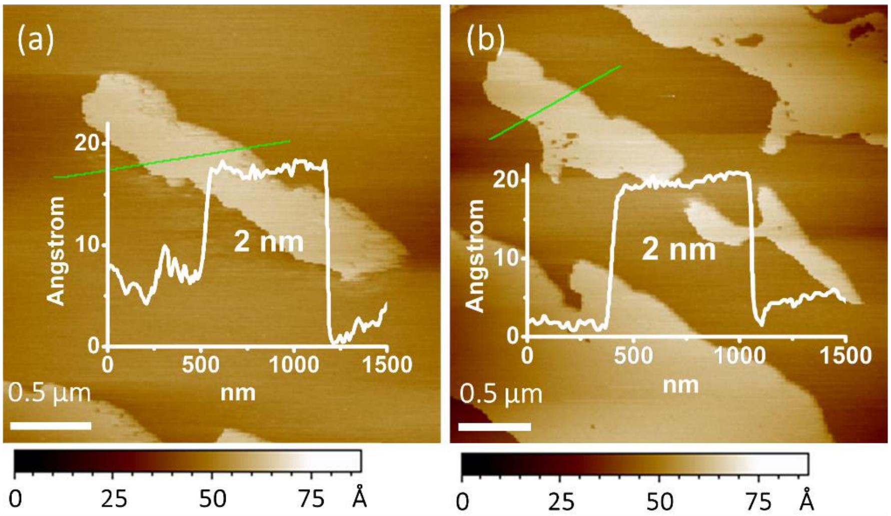

Figure S11. AFM image of Zr-BTB nanosheets (a) and PCN-134-2D (b) with the corresponding height profiles.

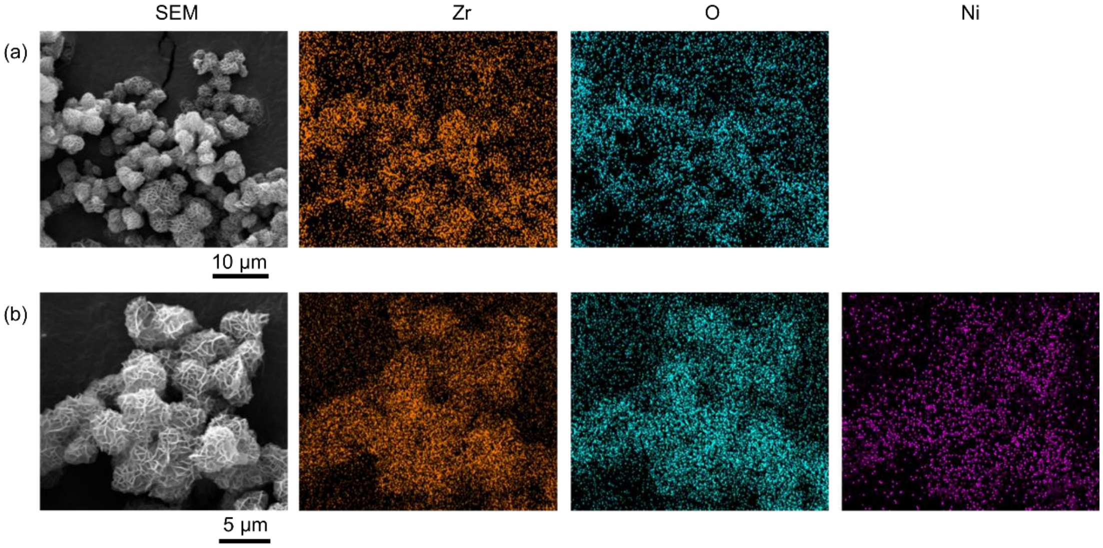

Figure S12. Elemental mapping by SEM/EDX of Zr-BTB before (a) and after (b) treatment with TCPP-Ni. Zr-BTB samples were synthesized using BA with 0.5 mL H_2O. The porphyrin centers were pre-occupied by Ni.

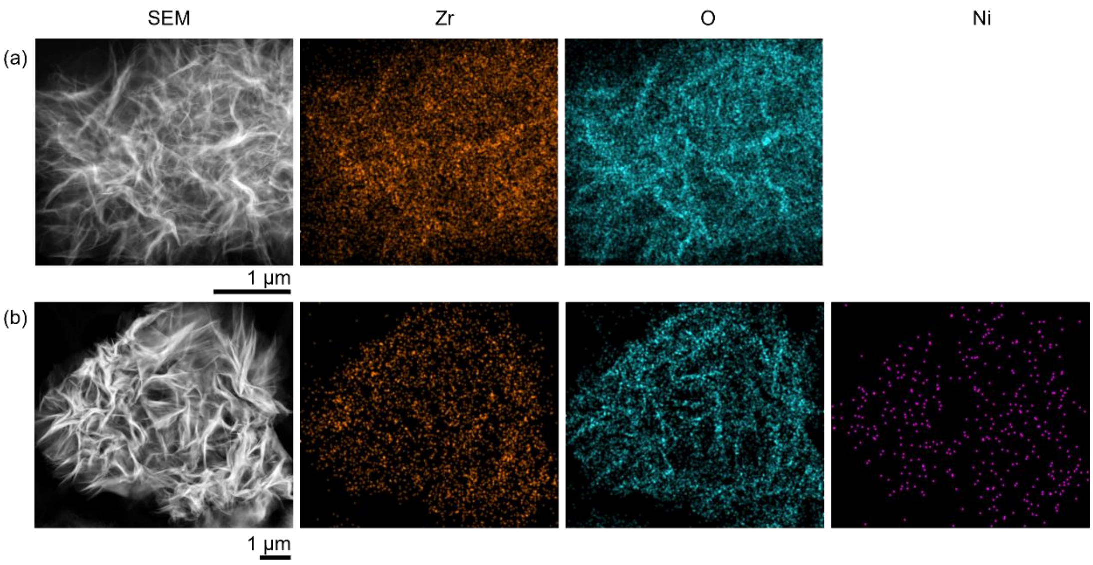

Figure S13. Elemental mapping by TEM/EDX of Zr-BTB before (a) and after (b) treatment with TCPP-Ni. Zr-BTB samples were synthesized using BA with 0.5 mL H_2O. The porphyrin centers are pre-occupied by Ni.

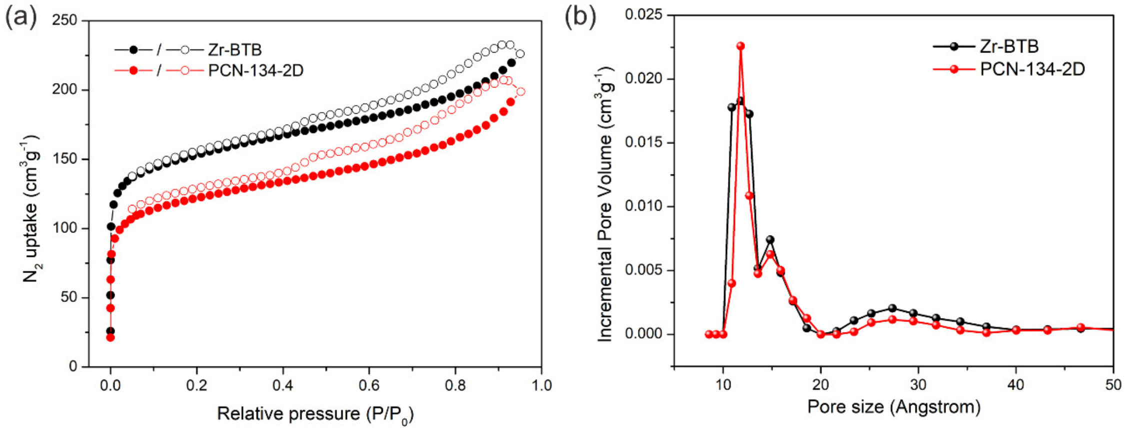

Figure S14. (a) N_2 sorption isotherms of Zr-BTB and PCN-134-2D at 77 K. (b) Pore size distributions of Zr-BTB and PCN-134-2D derived from adsorption branches of isotherms.

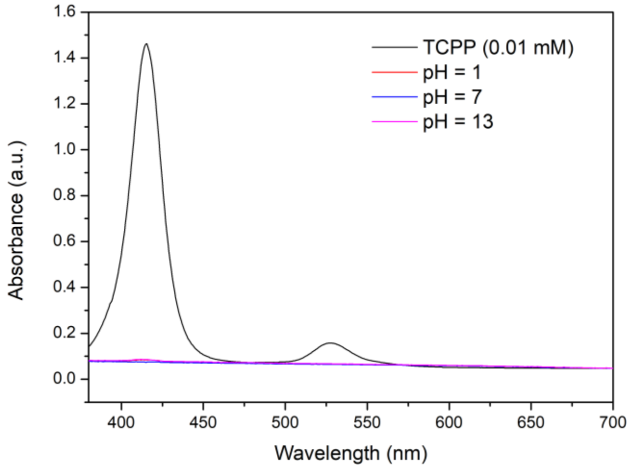

Figure S15. UV-vis spectra of supernatants after the stability test of PCN-134-2D indicating no TCPP leaching.

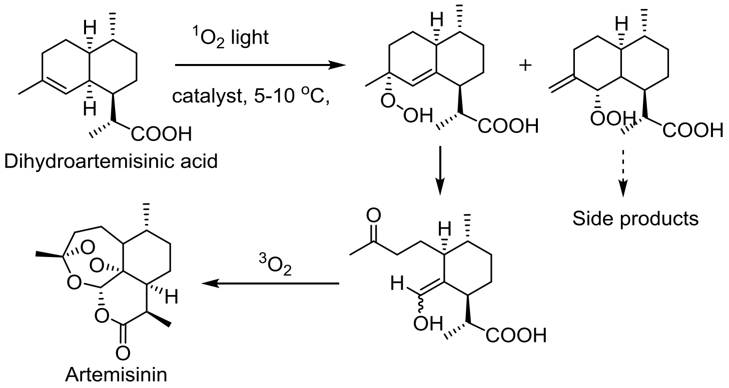

Figure S16. Reaction mechanism of photocatalytic oxidation of dihydroartemisinic acid to artemisinin.

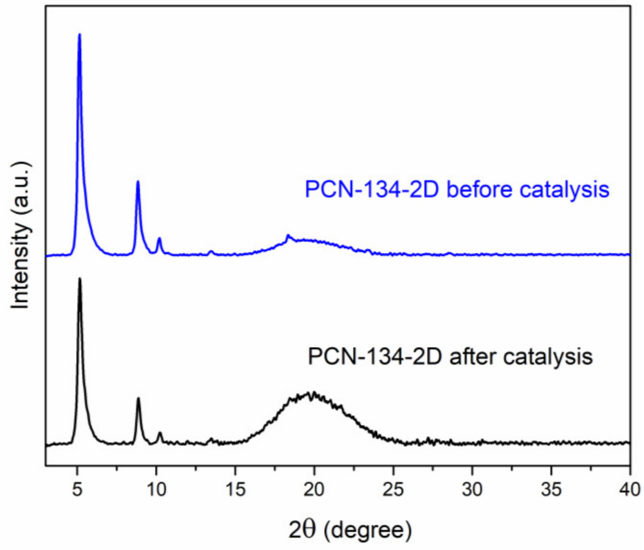

Figure S17. PXRD patterns of PCN-134-2D before and after catalysis.

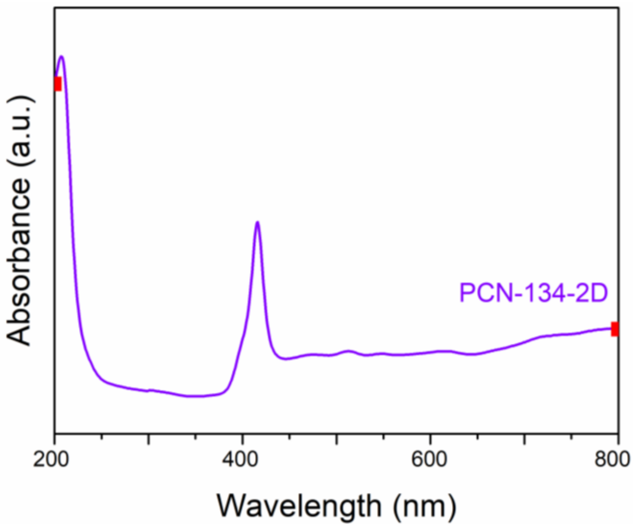

Figure S18. UV-vis spectra of PCN-134-2D in CH_3CN.

Table S1. Reaction conditions for the synthesis of Zr-BTB nanosheets.

**Entry**	**$ZrCl_4$/mg**	**H_3BTB/mg**	**DMF/mL**	**Acid**	**H_2O/mL**
**1**	10	10	3	Formic acid/1 mL	0
**2**	10	10	3	Formic acid/1 mL	0.25
**3**	10	10	3	Formic acid/1 mL	0.5
**4**	10	10	3	Acetic acid/1 mL	0
**5**	10	10	3	Acetic acid/1 mL	0.25
**6**	10	10	3	Acetic acid/1 mL	0.5
**7**	10	10	3	Propanoic acid/1 mL	0
**8**	10	10	3	Propanoic acid/1 mL	0.25
**9**	10	10	3	Propanoic acid/1 mL	0.5
**10**	10	10	3	Caproic acid/1 mL	0
**11**	10	10	3	Caproic acid/1 mL	0.25
**12**	10	10	3	Caproic acid/1 mL	0.5
**13**	10	10	3	Benzoic acid/0.6 g	0
**14**	10	10	3	Benzoic acid/0.6 g	0.25
**15**	10	10	3	Benzoic acid/0.6 g	0.5

Supplementary Methods

Materials and Instrumentation. All the reagents and solvents were commercially available and used as received. Powder X-ray diffraction (PXRD) was carried out with a Bruker D8-Focus Bragg-Brentano X-ray Powder Diffractometer equipped with a Cu sealed tube (λ = 1.54178 Å) at 40 kV and 40 mA. Nuclear magnetic resonance (NMR) data were collected on a Mercury 500 MHz spectrometer. The UV-vis absorption spectra were recorded on a Shimadzu UV-2450 spectrophotometer. Scanning electron microscope (SEM) was performed on FEI Quanta 600 FE. TEM was performed on FEI Tecnai G2 F20 ST FE. High resolution transmission electron microscope with FEG was performed on FEI Talos F200X. AFM measurements were performed with a PicoPlus in tapping mode (Agilent, US). TGA was carried out with a Mettler-Toledo DGA/DSC1 equipped with a gas controller for inert gas purging.

Synthesis of H_4TCPP. In a 500-mL three necked flask, methyl p-formylbenzoate (6.9 g, 0.042 mol) was dissolved in propionic acid (100 mL). Pyrrole was then added dropwise (3.0 mL, 0.043 mol) and the solution was refluxed for 12 h. After the reaction, the mixture was cooled to room temperature, and the precipitate was collected by suction-filtration and washed with methanol, ethyl acetate, and THF. After drying in an oven for 12 h, 1.9 g purple solid of TPPCOOMe (2.24 mmol, yield 21%) was obtained as a pure product. ^{1}H NMR (300 MHz, $CDCl_3$) δ 8.81 (s, 8H), 8.43 (d, 8H), 8.28 (d, 8H), 4.11 (s, 12H), 2.83 (s, 2H). The obtained ester (TPPCOOMe, 0.75 g) was stirred in 50 mL mixture of THF and MeOH (v:v = 1:1), to which a solution of NaOH (2.40 g, 60.00 mmol) in H_2O (25 mL) was introduced. This mixture was refluxed for 12 h. After cooling to room temperature, THF and MeOH were evaporated. Additional water was then added to the resulting water phase and the mixture was heated until the solid was fully dissolved. Then the homogeneous solution was acidified with 1 M HCl until no further precipitate was detected. The precipitate was collected by filtration, washed with water and dried in vacuum oven. FT/IR (KBr, cm^{-1}): v = 3444 (m), 3034 (w), 2634 (w), 1702 (s), 1614 (s), 1570 (m), 1404 (s), 1311 (m), 1277 (s), 1204 (m), 1180 (m), 1106 (m), 1004 (s), 862 (m), 799 (s), 770 (s), 721 (m).

Synthesis of H_4TCPP-Ni. A solution of TPPCOOMe (0.854 g, 1.0 mmol) and $NiCl_2 \cdot 6H_2O$ (3.1 g, 12.8 mmol) in 100 mL of DMF was refluxed for 6 h. After the mixture was cooled to room temperature, 150 mL of H_2O was added. The resultant precipitate was filtered and washed with 50 mL of H_2O twice. The obtained solid was dissolved in $CHCl_3$ and washed three times with 1 M HCl and twice with water. The

organic layer was dried over anhydrous magnesium sulfate and evaporated to afford quantitative crimson crystals. The obtained ester (0.75 g) was stirred in a THF (25 mL) and MeOH (25 mL) solvent mixture and a solution of KOH (2.63 g, 46.95 mmol) in H_2O (25 mL) was introduced. This mixture was refluxed for 12 h. After cooling down to room temperature, the THF and MeOH were evaporated. Additional water was added to the resulting water phase and the mixture was heated until the solid was fully dissolved, then the homogeneous solution was acidified with 1 M HCl until no further precipitate was detected. The crimson solid was collected by filtration, washed with water and dried in vacuum. FT/IR (KBr, cm^{-1}): ν = 3434 (m), 3034 (m), 2790 (w), 1692 (s), 1609 (s), 1541 (m), 1389(s), 1346 (m), 1311 (m), 1282 (s), 1175 (m), 1106 (m), 1004 (s), 867 (m), 833 (m), 799 (s), 716 (m) cm^{-1}.

Synthesis of Zr-BTB bulk material. $ZrCl_4$ (100 mg), H_3BTB (100 mg), benzoic acid (6 g) and DMF (30 mL) were charged in a Pyrex vial. The mixture was heated in 120 ºC oven for 48 h. After cooling down to room temperature, the product was collected by centrifugation, and washed with DMF twice and acetone twice (95 mg, yield: 46%). Calcd for $[Zr_6O_4(OH)_4](BTB)_2(BA)_6$: C, 50.64; H, 2.83%. Found: C, 50.13; H, 4.34%.

Synthesis of Zr-BTB nanosheets. $ZrCl_4$ (100 mg), H_3BTB (100 mg), benzoic acid (6 g), water 5 mL and DMF (30 mL) were charged in a Pyrex vial. The mixture was heated in 120 ºC oven for 48 h. After cooling down to room temperature, the product was collected by centrifugation, and washed with DMF twice and acetone twice (120 mg, yield: 59%). Calcd for $[Zr_6O_4(OH)_4](BTB)_2(BA)_3(OH)_3(H_2O)_3$: C, 44.62; H, 2.89%. Found: C, 45.55; H, 4.03%.

Synthesis of PCN-134-2D. Zr-BTB nanosheets (100 mg) were incubated in a solution of H_4TCPP in DMF (11 mM, 20 mL) at 100 ºC for 12 h. The solid was collected by centrifugation, and washed with DMF twice and acetone twice (115 mg, yield: 84%). The TCPP/BTB ratio was determined to be 0.46 by ^{1}H-NMR acid digestion experiments. The substitution of TCPP by TCPP-Ni under similar conditions yielded PCN-134-2D-Ni, which was used for SEM/EDX and TEM/EDX analysis. Calcd for $[Zr_6O_4(OH)_4](OH)_{4.2}(H_2O)_{4.2}(BTB)_2(H_2TCPP)_{0.9}$: C, 48.46; N, 2.09; H, 3.08%. Found: C, 45.55; N, 5.11; H, 4.33%.

Synthesis of PCN-134-3D. A mixture of $ZrCl_4$ (150 mg), BTB (50 mg) and DMF (10 mL) was charged in a Pyrex vial and heated in 85 ºC oven for 1 h. TCPP (30 mg) and BA (2.5 g) was dissolved in DMF (5 mL) and then added to the vial. The mixture was heated in 120 ºC oven for 24 h. After cooling down to room temperature, the red

crystals of PCN-134 were harvested (51 mg, yield: 57 %). The TCPP/BTB ratio was determined to be 0.33 by ^{1}H-NMR acid digestion experiments.

Thickness control of Zr-BTB nanosheets. The thickness of Zr-BTB nanosheets were controlled by adding different type of modulating acid and various amount of water to the reaction mixtures. Generally, a mixture of $ZrCl_4$ (10 mg), BTB (10 mg), DMF (3 mL), acid, and H_2O was charged in a Pyrex vial and heated in 120 ^{o}C oven for 48 h. After cooling down to room temperature, the solid was collected by centrifugation, and washed with DMF twice and acetone twice. The samples were analyzed by PXRD and SEM. The detailed synthetic conditions were summarized in Table S1.

^{1}H-NMR measurements. The TCPP/BTB ratio in each sample was determined by ^{1}H-NMR of digested samples. To digest samples for ^{1}H-NMR measurements, around 5 mg of samples were dissolved in 1 mL of a 10% D_2SO_4/DMSO-d_6. The ^{1}H-NMR spectra were collected on a Mercury 500 MHz spectrometer.

Gas adsorption measurements. Gas adsorption measurements were conducted using a Micrometritics ASAP 2020 system. Before gas sorption experiment, MOF samples was washed with DMF and exchanged with acetone for 3 days, during which the solvent was decanted and replenished with fresh solvent each day. The solid was dried under vacuum at 100 ^{o}C for 10 h, yielding a porous material.

Thermogravimetric analysis. For thermogravimetric analysis, about 10 mg of the sample was heated on a TGA/DSC1 (Mettler-Toledo) thermogravimetric analyzer from room temperature to 700 °C at a rate of 5 $°C\cdot min^{-1}$ under N_2 flow of 50 $mL\cdot min^{-1}$. The initial weight loss before 300 °C is attributed to the removal of the coordinated water molecules on the clusters, which is comparable to the theoretical mass percentage. The TGA curves indicate thermal stability of the framework up to 450 °C, where decomposition of the framework begins.

Photocatalytic $^{1}O_2$ generation. The $^{1}O_2$ generation was monitored by the $^{1}O_2$ scavenger, 1,3-diphenylisobenzofuran (DPBF), using UV-Vis. A halogen lamp with a 455 nm long pass filter was used as the light source. Acetonitrile (50 mL) was bubbled with oxygen for 20 minutes before the measurements. MOF catalyst (1 μM based on the porphyrin loading) was added to the DPBF solution in acetonitrile (70 μM) and bubbled with oxygen under visible light irradiation. The samples were taken at different time intervals for UV-Vis measurements.

Photocatalytic oxidation of dihydroartemisinic acid to artemisinin. The synthesis of artemisinin was conducted using PCN-134-2D nanosheets as photocatalyst under

visible light in the presence of O_2. In a typical reaction, dihydroartemisinic acid (25 mg, 0.106 mmol), MOF catalysts (0.002 mmol based on porphyrin) and trifluoroacetic acid (8 μL) were dispersed in dichloromethane (5 mL) or EtOH:H_2O (1:1, 5mL) and slowly bubbled with O_2 under the irradiation of LED lamps (150 W) at 5-10°C for 3 hours. After removal of dichloromethane, conversion and selectivity to artemisinin was measured by ^{1}H-NMR in $CDCl_3$ using biphenyl (16.3 mg, 0.106 mmol) as an internal standard.
